# Low-Temperature Processed TiO_x_ Electron Transport Layer for Efficient Planar Perovskite Solar Cells

**DOI:** 10.3390/nano10091676

**Published:** 2020-08-26

**Authors:** Md. Shahiduzzaman, Daiki Kuwahara, Masahiro Nakano, Makoto Karakawa, Kohshin Takahashi, Jean-Michel Nunzi, Tetsuya Taima

**Affiliations:** 1 Nanomaterials Research Institute, Kanazawa University, Kakuma, Kanazawa 920-1192, Japan; karakawa@staff.kanazawa-u.ac.jp (M.K.); nunzijm@queensu.ca (J.-M.N.); 2 Graduate School of Natural Science and Technology, Kanazawa University, Kakuma, Kanazawa 920-1192, Japan; kuworld.shining.0626@gmail.com (D.K.); masahiro-nakano@se.kanazawa-u.ac.jp (M.N.); ktakaha@kvj.biglobe.ne.jp (K.T.); 3 Graduate School of Frontier Science Initiative, Kanazawa University, Kakuma, Kanazawa 920-1192, Japan; 4 Department of Physics, Engineering Physics and Astronomy, Queen’s University, Kingston, ON K7L 3N6, Canada

**Keywords:** low-temperature TiO_x_ layer, high-temperature TiO_2_ layer, perovskite solar cells

## Abstract

The most frequently used n-type electron transport layer (ETL) in high-efficiency perovskite solar cells (PSCs) is based on titanium oxide (TiO_2_) films, involving a high-temperature sintering (>450 °C) process. In this work, a dense, uniform, and pinhole-free compact titanium dioxide (TiO_x_) film was prepared via a facile chemical bath deposition process at a low temperature (80 °C), and was applied as a high-quality ETL for efficient planar PSCs. We tested and compared as-deposited substrates sintered at low temperatures (< 150 °C) and high temperatures (> 450 °C), as well as their corresponding photovoltaic properties. PSCs with a high-temperature treated TiO_2_ compact layer (CL) exhibited power conversion efficiencies (PCEs) as high as 15.50%, which was close to those of PSCs with low-temperature treated TiO_x_ (14.51%). This indicates that low-temperature treated TiO_x_ can be a potential ETL candidate for planar PSCs. In summary, this work reports on the fabrication of low-temperature processed PSCs, and can be of interest for the design and fabrication of future low-cost and flexible solar modules.

## 1. Introduction

Solid-state organic–inorganic hybrid perovskite solar cells (PSCs) have been one of the most significant discoveries in the field of photovoltaics because of their advantages, including a low-cost device fabrication process, desirable energy harvesting characteristics, light weight, and flexibility [1,2,3,4]. The first report of PSCs with a power conversation efficiency (PCE) of 3.8% was by Miyasaka et al. in 2009 [5]. Intensive research efforts have subsequently been undertaken on PSCs, with a reported record PCE of 25.2% under laboratory conditions [6]. In conventional PSC architecture, a perovskite film is sandwiched in between an electron transport layer (ETL) and a hole transport layer (HTL), and both layers are also sandwiched between a transparent electrode and a metal electrode in order to fabricate complete devices. In terms of improving PSC performance, it is significantly important to produce a uniform, compact, pinhole-free, and full surface coverage titanium oxide (TiO_2_) compact layer (CL) as the ETL in order to achieve more efficient electron transport, charge extraction, and low interfacial recombination [7]. PSCs can be fabricated entirely using low-cost solution processing [8] and vacuum deposition methods [9,10]. The high stability and efficiency of PSCs with low-cost processing can enable the realization of economically competitive solar power. In general, there are two leading device configurations for PSCs: planar heterojunction (PHJ) and mesoporous scaffold-based devices [11,12,13]. Thin ETLs serve a key function of extracting electrons from the perovskite layer, and blocking recombination between electrons in the fluorine-doped tin oxide (FTO) and holes in the perovskite layer. A thick ETL can minimize recombination; however, the electron flow may be limited because of high series resistance. Very few PSC structures have been fabricated without an ETL, and they generally have low PCEs compared with PHJ PSCs prepared with an ETL [14,15]. High-quality mesoporous TiO_2_ scaffold films and TiO_2_ CL require a high-temperature sintering (> 450 °C) step, which limits their applications in future flexible PSCs and solar modules. The active surface area and performance of the devices was improved by forming the perovskite layer on a layer composed of TiO_2_ CL/TiO_2_ nanoparticles [16,17,18]. The development of PHJ PSCs with low-temperature processed (< 150 °C) ETL CLs has attracted significant attention for their simple device framework [19].

Several groups have developed ETLs using a TiO_2_ CL with different deposition techniques, including spin coating [20], spray pyrolysis [21,22,23], oblique electrostatic inkjet [24], electrodeposition [25], inkjet printing [26], atomic layer deposition [27], sputtering [28], and chemical bath deposition (CBD) [29] for PSC applications. Notable ETLs can be fabricated using either spin coating or spray pyrolysis top-down techniques, although these are quite sensitive to the control parameters, and subsequently the PCE of such fabricated devices may vary considerably. Alberti et al. reported a low-temperature nanostructured TiO_2_ layer via reactive sputtering, with a maximum PCE of ∼15% without surface treatments or additional layers [28]. Sputtering demands a vacuum environment and has a slow deposition rate that presents challenges for producing TiO_2_ films. However, unlike other bottom-up techniques, the CBD technique is an encouraging candidate for producing TiO_2_ CLs using a low-cost fabrication process that is compatible with large-scale fabrication. The CBD technique produces high-quality TiO_2_ CLs, which can be used as the ETL in PSCs. However, Takahashi and coworkers [30] developed a simple, low-cost, uniform, air-stable, scalable CBD technique that was followed by low-temperature thermal annealing (< 150 °C) in order to deposit titanium dioxide (TiO_x_) CLs with controlled film thicknesses for the deposition of the ETL in organic solar cells. In addition, Chen and coworkers reported a relatively low-temperature processed flower-like TiO_2_ ETL-based PSC with a PCE of 15.71% [31]. Furthermore, Liu et al. [32] prepared Nb-doped TiO_2_ via a CBD, and achieved a PCE as high as 19.23%. However, a fundamental understanding of the CBD growth of the TiO_2_ films has not been studied in depth in order to realize their full potential. Therefore, it is important to develop PHJ PSCs with low-temperature processed (< 150 °C) CBD–TiO_x_ CL ETLs in order to have the possibility of fabricating cost-effective solar modules. Moreover, low-temperature processing is mandatory for the development of flexible solar cells and modules [33,34].

In this study, we used a simple, low-temperature, scalable CBD process to fabricate a uniform, pinhole free, and air stable amorphous TiO_x_ film. We investigated and compared the formation of compact TiO_x_ processed at a low temperature (< 150 °C) and compact TiO_2_ treated at a high temperature (> 450 °C) as ETLs, as well as their corresponding solar cell properties. While there was a difference in crystallinity between the TiO_x_ and TiO_2_ films, no difference was observed in the chemical bonding states and surface morphology. In addition, the perovskite films formed on the TiO_x_ and TiO_2_ films had similar crystallinities and surface morphologies, and their device performances were comparable.

## 2. Materials and Methods

### 2.1. Materials

All of the commercially available materials were purchased and used as received, without further purification. Fluorine-doped tin oxide (FTO) and indium tin oxide (ITO)-patterned glass substrates were purchased from Asahi Glass (Tokyo, Japan). Lead iodide (PbI_2_; purity 98%) and methylammonium iodide (CH_3_NH_3_I; purity 98%) were purchased from Tokyo Chemical Industry (Tokyo, Japan). Titanium (IV) oxysulfate (TiOSO_4_; purity 99.99%) was purchased from Sigma Aldrich (St. Louis, MO, USA). Hydrogen peroxide (H_2_O_2_; purity 35%) and *N, N*-dimethylformamide (DMF; purity 99.5%) were supplied by Kanto Chemical (Tokyo, Japan).

### 2.2. Device Fabrication

FTO- and ITO-patterned glass substrates were sequentially cleaned in a sonication bath with a KOH solution (1.4 g of KOH dissolved in 50 mL of ultrapure water) followed by ultrapure water for 10 min each for two cycles. The cleaned FTO and ITO substrates were then dried with nitrogen flow and pre-treated using oxygen plasma for 20 min prior to use. A complex precursor solution was prepared by adding water as a solvent, TiOSO_4_ as a titanium source, and H_2_O_2_ as a complexing agent, followed by heating, and was deposited on FTO or ITO substrates, according to the procedure described by Kuwabara et al. [30]. Then, the as-deposited substrates were baked at a low temperature (<150 °C) for 1 h and at a high temperature (> 450 °C) for 30 min. Then, 0.482 g of PbI_2_ and 0.168 g of CH_3_NH_3_I were dissolved and mixed in anhydrous DMF (652 µL):DMSO (163 µL) at a ratio of 4:1. The perovskite precursor solution was stirred at 70 °C for 60 min prior to spin coating. A perovskite precursor solution was spin-coated in three steps, as follows: first step, 0 rpm for 10 s; second step, 1000 rpm for 10 s; and third step, 5000 rpm for 30 s. In the third step, 400 µL of chlorobenzene solvent was dripped on the substrate 5 s before the end of spin-coating, and was transferred to a hot plate at 100 °C for 60 min in a glove box under an inert environment and then cooled to room temperature. The Spiro-OMeTAD solution, used as the hole transport layer, was prepared according to the report by Wakamiya et al. [35]. Finally, a gold (Au) electrode with a thickness of 100 nm was deposited at a 1.0 Å/s growth rate on the Spiro-OMeTAD layer so as to complete the device.

### 2.3. Characterization

Scanning electron microscopy (SEMSU1510, Hitachi High-Tech, Tokyo, Japan) together with atomic force microscopy (AFM; SII SPI3800N, Seiko, Japan) were used to analyze the surface morphology. The X-ray diffraction (XRD) patterns of the prepared films were measured using an X-ray diffractometer (SmartLab, Rigaku, Japan) with an X-ray tube (Cu Kα radiation, λ = 1.5406 Å). Surfcorder (ET 200, Tokyo, Japan) was used to measure the thickness of the films. The current density versus voltage (J–V) characteristics of all of the fabricated PSCs were measured at a scan rate of 0.05 V/s in forward scan (FS; from −0.2 to 1.2 V) and reverse scan (RS; from 1.2 to −0.2 V) directions. Each device had a 0.09 cm^2^ active area. Measurements were obtained under 100 mW/cm^2^ AM 1.5G irradiation from a solar simulator, and were measured using a Keithley 2401 digital source meter. The incident photon-to-conversion efficiency (IPCE) spectrum of each device was measured using a monochromatic xenon arc light system (Bunkoukeiki, SMI-250JA, Tokyo, Japan).

## 3. Results and Discussion

**Figure 1:**
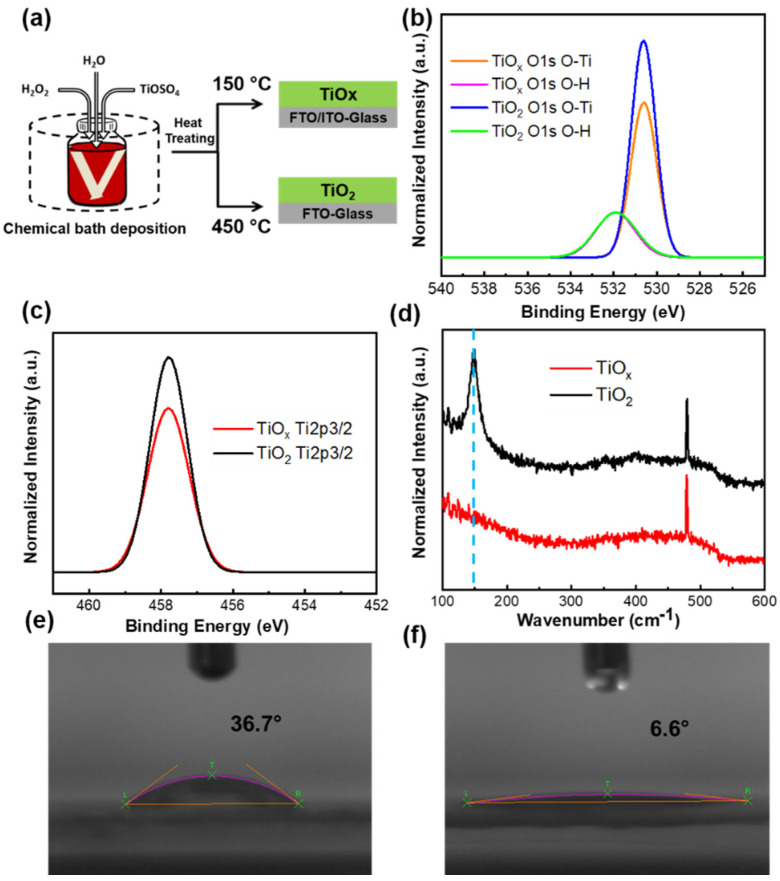
(**a**) Schematic illustration of the chemical bath deposited titanium dioxide (TiOx) films, as well as their corresponding thermal treatments at 150 °C for 1 h and 450 °C for 30 min; XPS spectra of TiO_x_ and titanium oxide (TiO_2_), (**b**) O1s, and (**c**) Ti2p; (**d**) Raman spectra of TiO_x_ and TiO_2_ films; and water contact angles of the (**e**) TiO_x_ and (**f**) TiO_2_ films on an FTO substrate.

Figure 1a shows a schematic of the facile chemical bath deposition process at a low temperature (80 °C) for fabrication of the TiO_x_ films, as well as their corresponding thermal treatments at 150 °C for 1 h and 450 °C for 30 min. Figure 1b,c shows the XPS spectra of the TiO_x_ and TiO_2_ films. The Ti2p peak positions were unchanged for both the low-temperature processed TiO_x_ and high-temperature treated TiO_2_ films, indicating that the TiO_x_ showed the same chemical bonding state as TiO_2_ (Figure 1c). In addition, from the XPS spectrum, the peak intensity of TiO_2_ O1s O–Ti and Ti2p3/2 increased compared with TiO_x_, as shown in Figure 1b,c. This peak can be attributed to the high crystallinity of the TiO_2_ film; the high-temperature treated TiO_2_ retained its crystallinity, while the low-temperature TiO_x_ had an amorphous structure (Figure S1). The obtained Raman spectra further confirmed these results. A peak for the anatase crystal structure of the TiO_2_ film was confirmed at ~150 cm^−1^, while no peak (black line) was found in the TiO_x_ film (Figure 1d). This observation is similar to those previously reported by Sangaletti et al. [36]. Therefore, it can be inferred that the bond strength of TiO_2_ changed because of the crystallinity.

Water droplets on the TiO_x_ and TiO_2_ films are shown in Figure 1e,f. The TiO_x_ film shows a higher water contact angle of 36.7°, while the TiO_2_ film exhibits a lower contact angle of 6.6°, indicating that the TiO_x_ film had a lower wettability compared with the TiO_2_ film. The superior wettability of the TiO_2_ film may be another avenue to improve the PCE of the resulting PSCs (as discussed later). In addition, we performed contact angle measurements to evaluate the surface-free energy of the TiO_x_ and TiO_2_ films. The contact angles at three different points on each film surface with water, formamide, and ethylene glycol were obtained and the average values were used to calculate the surface-free energy of each film [37]. The surface-free energy of the TiO_x_ and TiO_2_ films was 56.9 mJm^−2^ and 51.8 mJm^−2^, respectively, suggesting that a lower surface-free energy facilitates the formation of a more stable film surface. Therefore, it can be concluded that the surface state of the high-temperature treated TiO_2_ film affords a higher stability than the low-temperature treated TiO_x_ film.

**Figure 2:**
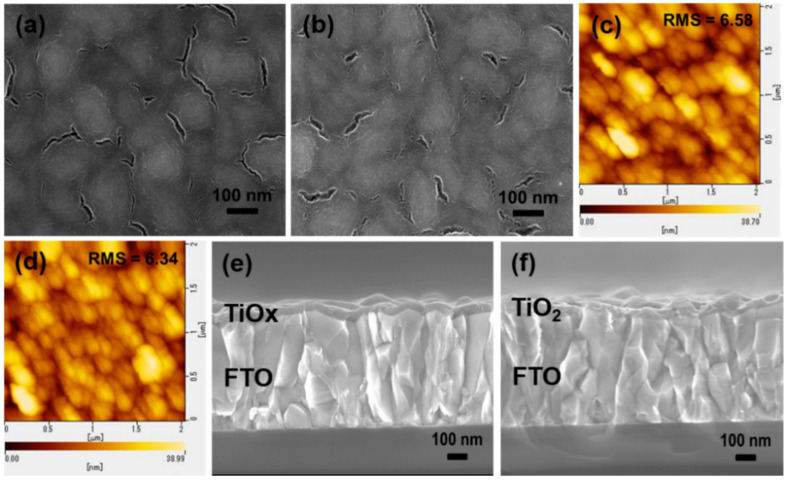
SEM images of the (**a**) TiO_x_ film and (**b**) TiO_2_ film; atomic force microscopy (AFM) images of the (**c**) TiO_x_ film and (**d**) TiO_2_ film; and cross-section SEM images of the (**e**) TiO_x_ film and (**f**) TiO_2_ film.

Top-view SEM images of the TiO_x_ and TiO_2_ films are shown in Figure 2a,b. It can be seen that almost similar surface morphologies that are uniform, dense, and pinhole free on an FTO substrate were obtained with both films. These smooth, pinhole-free, and dense scaffolds may offer efficient charge extraction and hole blocking in the resulting PSCs. This was further confirmed via atomic force microscopy (AFM) analysis, as shown in Figure 2c,d. The root mean square (RMS) roughness for the TiO_x_ and TiO_2_ films was 6.58 and 6.34 nm, respectively, indicating that the film surface roughness was nearly equivalent in both cases. It can be concluded for both samples that the surface morphology and roughness showed similar trends, regardless of the crystallinity. Cross-sectional SEM images of the TiO_x_ and TiO_2_ films are shown in Figure 2e,f. Both samples clearly show smooth films deposited (60 nm thickness) on the FTO substrates, which efficiently blocked direct contact between the FTO and perovskites. This implies that low-temperature treated TiO_x_ solely serves as a potential ETL candidate for planar PSCs.

**Figure 3:**
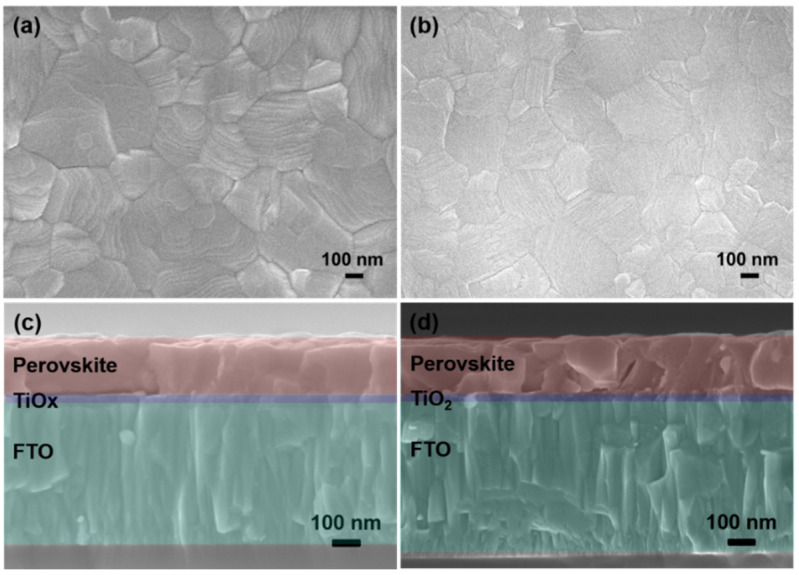
Top-view SEM images of the (**a**) TiO_x_/MAPbI3 film and (**b**) TiO_2_/MAPbI_3_ film, and cross-sectional SEM images of the (**c**) FTO/TiO_x_/MAPbI_3_ film and (**d**) FTO/TiO_2_/MAPbI_3_ film.

Figure 3a,b shows the top-view SEM images of the TiO_x_/MAPbI_3_ and TiO_2_/MAPbI_3_ films. It can be seen that both samples had large crystal grains with a uniform and flat surface morphology. The film with a smooth surface morphology and large perovskite grains has fewer grain boundaries and fewer traps, which aids in reducing the charge carrier losses at the trap states in the grain boundaries [38]. We compared the cross-sectional SEM images of the TiO_x_- and TiO_2_-based PSCs, as shown in Figure 3c,d, respectively. Both samples had perovskite films with similar thicknesses of 300 nm.

**Figure 4:**
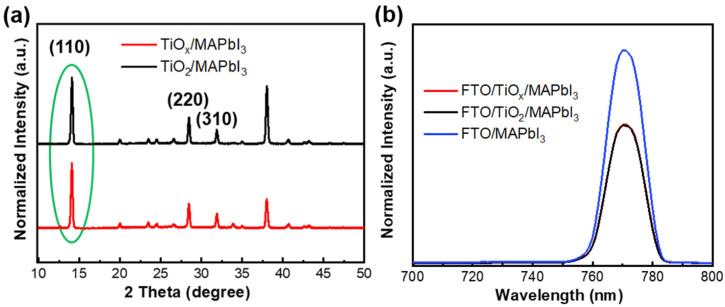
(**a**) XRD pattern of the perovskites formed on the TiO_x_ and TiO_2_ films, and (**b**) photoluminescence (PL) spectra of the perovskites formed on the FTO substrate, FTO/TiO_x_ film, and FTO/TiO_2_ film.

The XRD patterns of the TiO_x_/MAPbI_3_ and TiO_2_/MAPbI_3_ films are shown in Figure 4a,b. Diffraction peaks were detected at 2θ angles of 14.1°, 28.5°, and 31.8° in the TiO_x_/MAPbI_3_ and TiO_2_/MAPbI_3_ perovskite films, and the peaks were assigned to the (110), (220), and (310) crystal planes, respectively. There was no peak from PbI_2_ at 12.6° for both samples, indicating the complete transformation of PbI_2_. The full width at half maximum (FWHM) of the TiO_x_/MAPbI_3_ and TiO_2_/MAPbI_3_ films was 0.246 and 0.247, respectively, suggesting a similar crystallinity for the perovskite films, despite the difference in crystallinity for their respective ETLs [39]. The photoluminescence (PL) spectra of the FTO/MAPbI_3_, FTO/TiO_x_/MAPbI_3_, and FTO/TiO_2_/MAPbI_3_ films were measured and the results are shown in Figure 4b. The peak intensity was lower, in addition to an increased PL quenching compared with the perovskite formed on the FTO substrate, which confirms that efficient charge extraction occurred from the perovskite film. When the perovskite films were formed on TiO_x_ and TiO_2_, a similar PL quenching was obtained for both samples, which implied efficient electron transfer from the perovskite film to the ETLs. This suggests that the TiO_x_ and TiO_2_ films have similar charge extraction capabilities [40,41].

**Figure 5:**
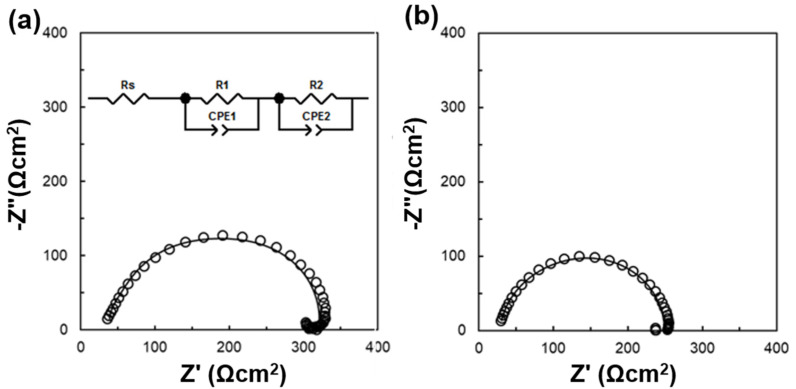
Nyquist plots of perovskite solar cells (PSCs) based on (**a**) TiOx and (**b**) TiO_2_ in the dark at a DC bias of 0 V. A 1–V AC signal was applied with a frequency range of 20 Hz–1 MHz. The inset shows the equivalent circuit model of the resultant devices.

We carried out electrochemical impedance spectroscopy (EIS) to further investigate the electrical properties of each interface, including the charge transfer, carrier recombination, and inner series resistance. Figure 5 shows Nyquist plots of the TiO_x_- and TiO_2_-based devices at zero bias in the dark and under AM 1.5G–100 mW/cm^2^ simulated sunlight irradiation. According to the equivalent circuit model shown in the Figure 5a inset, detailed Nyquist plots fitting the analysis parameters of the corresponding devices are summarized in Table S1. R1 can be considered a resistance component derived from the ETL, while R2 is termed as a resistance component derived from the interface between the ETL and perovskite. R1 is a resistance component derived from the ETL and is labeled as charge transferred resistance (RCT). The reduction in RCT contributed to the superior charge transfer in the TiO_2_-based device compared with the TiO_x_-based device, implying that a difference in the crystallinity of TiO_2_ may contribute to charge transfer at the interface. In addition, R2 is a resistance component at the interface and is considered to be a charge recombination resistance. The larger value contributed to a lower recombination at the interfaces. Mostly equivalent values were obtained for both devices for R2, which is consistent with the results from the corresponding PL spectra (Figure 4b).

**Figure 6:**
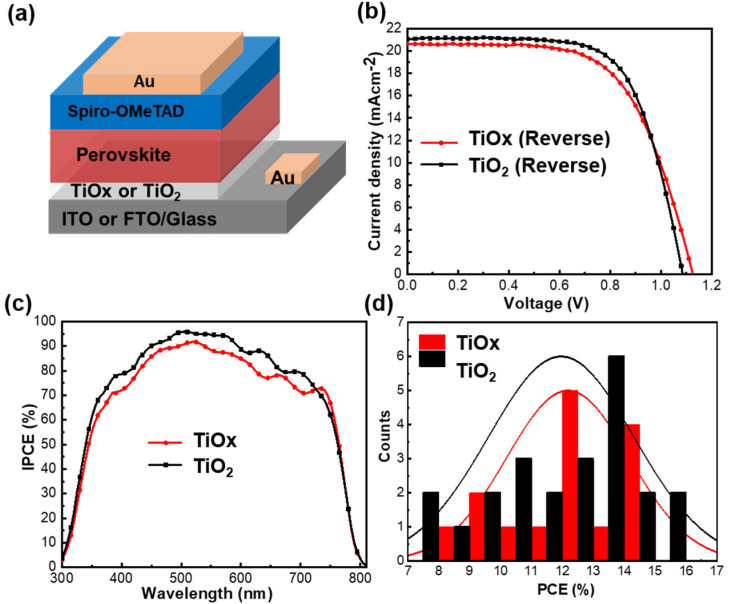
(**a**) Schematic of the complete device configuration, (**b**) reverse scan J–V curves of the TiO_x_- and TiO_2_-based PSCs, (**c**) incident photon-to-conversion efficiency (IPCE) spectra of the TiO_x_- and TiO_2_-based PSCs, and (**d**) histogram of the power conversation efficiencies (PCE) of PSCs with the TiO_x_ and TiO_2_ films.

Figure 6a shows the complete device structure. The current density versus voltage (J–V) characteristics under 1 sun AM 1.5G (100 mW/cm^2^) for the TiO_x_- and TiO_2_-based PSCs are shown in Figure 6b. The corresponding device parameters are summarized in Table 1. A comparison of the scan direction for the FS and RS is provided in Table S2. The PSC with the TiO_x_ film exhibited a short circuit current density (J_sc_) of 20.64 mAcm^−2^, open-circuit voltage (V_oc_) of 1.12 V, fill factor (FF) of 0.63, and PCE of 14.51% in the RS direction. The PSC with the TiO_2_ film had a J_sc_ of 21.06 mAcm^−2^, V_oc_ of 1.08, FF of 0.68, and PCE of 15.50% in the RS direction. The enhancement of J_sc_ and FF was attributed to a lowering of the injection barrier at the interface between the TiO_2_ CL and perovskite; this is because the high-temperature treated TiO_2_ CL formed a smooth interface (Figure 1e), which facilitated efficient electron flow. The enhancement of V_oc_ was unclear for the TiO_x_-based PSCs compared with the TiO_2_-based PSCs. Additionally, large hysteresis was observed in the J–V curves for both devices (Figure S2a). Previous reports have demonstrated a similar hysteresis behavior for TiO_2_ CL-based PSCs because of the rough interface between the TiO_2_ and perovskite [42]. The PSC with TiO_2_ CL as the ETL exhibited a PCE as high as 15.50%, which is close to that of the PSC with the TiO_x_ ETL (14.51%). This implies that thermal annealing at a high temperature is not necessary in order to achieve high-performance PSCs. The incident photon-to-conversion efficiency (IPCE) was measured in order to verify the reproducibility of the PSCs based on the TiO_x_ and TiO_2_ films, as shown in Figure 6c. The PSCs with TiO_x_ and TiO_2_ films produced integrated photocurrents of 19.1 and 20.02 mAcm^−2^, respectively. The IPCE value of the TiO_2_-based PSC was slightly higher than that of the TiO_x_-based PSC over the same wavelength range, indicating that the electrons were efficiently collected at the interface between the TiO_2_ CL and perovskite, along with a reduction in the interfacial energy barrier. A histogram of the device PCEs for both the TiO_x_- and TiO_2_-based devices is shown in Figure 6d. The PCE distributions for both devices were nearly similar, implying that a low-temperature processed TiO_x_ film can be used as an efficient ETL in future flexible solar modules.

**Figure 7:**
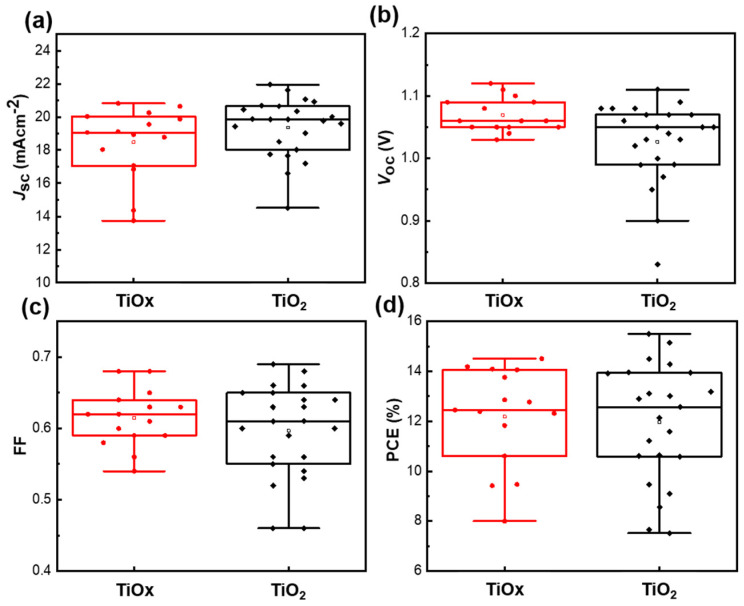
Average (**a**) *J*_sc_, (**b**) *V*_oc_, (**c**) fill factor (FF), and (**d**) PCE values for devices fabricated with TiO_x_ and TiO_2_ films. Error bars indicate ± one standard deviation from the mean.

To verify the reproducibility of the devices based on the TiO_x_ and TiO_2_ films, we compared the average J_sc_, V_oc_, FF, and PCE values of 18 and 26 individual devices, respectively, as shown in Figure 7. Reproducibility is one of the most important parameters for modularization. The low-temperature treated TiO_x_ and high-temperature treated TiO_2_ samples showed similar reproducibility results. This indicated that low-temperature treated TiO_x_ performs as well as the high-temperature treated TiO_2_ in PSCs. Furthermore, we investigated the effectiveness of the low-temperature (< 150 °C) processed TiO_x_ on an ITO substrate, which exhibited a satisfactory photovoltaic performance compared with the FTO substrate (Figure S2b). The corresponding PSC parameters are summarized in Table S3. It can be concluded that a similar trend was observed for the low-temperature treated TiO_x_ on an ITO substrate device. Therefore, the results show that the low-temperature treated TiO_x_ film had a beneficial contribution to the PSC devices, which could help in further lowering the cost of the module manufacturing process in the future.

## 4. Conclusions

In this work, we fabricated and compared as-deposited substrates with TiO_x_ and TiO_2_ films sintered at low temperatures (< 150 °C) and high temperatures (> 450 °C), and investigated their corresponding photovoltaic properties. The TiOx-based PSCs exhibited a satisfactory photovoltaic performance compared with the TiO_2_-based PSCs. The PSC with a TiO_x_ CL showed a PCE of 14.51%, which was very close to that of the TiO_2_ CL-based PSCs (15.50%). In addition, a similar reproducibility was observed for devices fabricated using the TiO_x_ and TiO_2_ films. This suggests that TiO_x_ CL serves as a potential ETL in the PSC. There was a difference in crystallinity between the TiO_x_ and TiO_2_ films, while the chemical bonding states and surface morphology were similar. Furthermore, there was no difference between the perovskite films grown on TiO_x_ and TiO_2_ films, and an almost similar device performance was achieved. This work can enable the fabrication of entirely low-temperature processed PSCs, and could possibly contribute to the fabrication of flexible solar modules in the future.

## Figures and Tables

**Table 1:** Summary of the parameters for the PSCs (FTO-glass/TiO_x_ or TiO_2_/MAPbI_3_/Spiro-OMeTAD/Au). The statistical analysis (average ± standard deviation) was based on the measurements of 18 and 26 individual devices with TiO_x_ and TiO_2_, respectively. Champion refers to the device with the highest PCE.

ETLs Layer		J_SC_ (mA/cm^2^)	V_oc_ (V)	FF	PCE (%)
TiO_x_	Champion	20.64	1.12	0.63	14.51
	Average ± SD	18.3 ± 2.3	1.07 ± 0.03	0.61 ± 0.04	12.1 ± 2.1
TiO_2_	Champion	21.06	1.08	0.68	15.50
	Average ± SD	19.2 ± 1.9	1.02 ± 0.07	0.59 ± 0.07	11.9 ± 2.4

## Supplementary Materials

The following are available online at https://www.mdpi.com/2079-4991/10/9/1676/s1: Figure S1: XRD pattern of low-temperature treated TiO_x_ film on an FTO substrate; Figure S2: Forward scan and reverse scan J–V curves of (a) TiO_x_ and TiO_2_ films deposited on an FTO substrate; (b) TiO_x_ film grown on an ITO substrate; Table S1: Nyquist plots fitting the analysis parameters; Table S2: Summary of device performance characteristics with TiO_x_- and TiO_2_-based PSCs; Table S3: Summary of device performance characteristics with a TiO_x_ film deposited on an ITO substrate-based PSC.
