# Supplementary material for: Low-Temperature Processed TiOx Electron Transport Layer for Efficient Planar Perovskite Solar Cells
Source: Nanomaterials (Basel). 2020 Aug 26;10(9):1676. doi: 10.3390/nano10091676 (PMC7559000; doi:10.3390/nano10091676)
Supplement: Supplementary file 1 [file nanomaterials-10-01676-s001.pdf]

Supporting Information

Low-temperature Processed TiO_x Electron Transport Layer for Efficient Planar Perovskite Solar Cells

Md. Shahiduzzaman [1,*], Daiki Kuwahara [2], Masahiro Nakano [2], Makoto Karakawa [1,2,3], Kohshin Takahashi [2], Jean-Michel Nunzi [1,4] and Tetsuya Taima [1,2,3,*]

1 Nanomaterials Research Institute, Kanazawa University, Kakuma, Kanazawa 920-1192, Japan; karakawa@staff.kanazawa-u.ac.jp (M.K.); nunzijm@queensu.ca (J.-M.N.)

2 Graduate School of Natural Science and Technology, Kanazawa University, Kakuma, Kanazawa 920-1192, Japan; kuworld.shining.0626@gmail.com (D.K.); masahiro-nakano@se.kanazawa-u.ac.jp (M.N.); ktakaha@kvj.biglobe.ne.jp (K.T.)

3 Graduate School of Frontier Science Initiative, Kanazawa University, Kakuma, Kanazawa 920-1192, Japan

4 Department of Physics, Engineering Physics and Astronomy, Queen's University, Kingston, ON K7L 3N6, Canada

***** Correspondence: shahiduzzaman@se.kanazawa-u.ac.jp (M.S.); taima@se.kanazawa-u.ac.jp (T.T.); Tel.: +81-76-234-4937 (M.S.)

Table of Contents

Figure S1. XRD pattern of low-temperature treated-TiO_x film on an FTO-substrate....S3

Figure S2: Forward Scan and Reverse Scan *J-V* curves of (a) TiO_x and TiO_2 films deposited on an FTO-substrate; (b) TiO_x film grown on ITO-substrate…………………………………………………………………….……..…S4

Table S1. Nyquist plots fitting analysis parameters……………………………………S5

Table S2. Summary of device performance characteristics with TiO_x and TiO_2 based PSCs. ....…...S6

Table S3. Summary of device performance characteristics with TiO_x film deposited on an ITO-substrate based PSCs….……………………………….……..………..…...S7

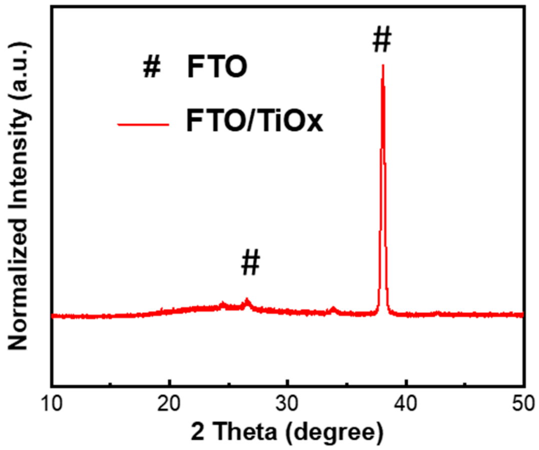

Figure S1. XRD pattern of low-temperature treated-TiOx film on FTO-substrate.

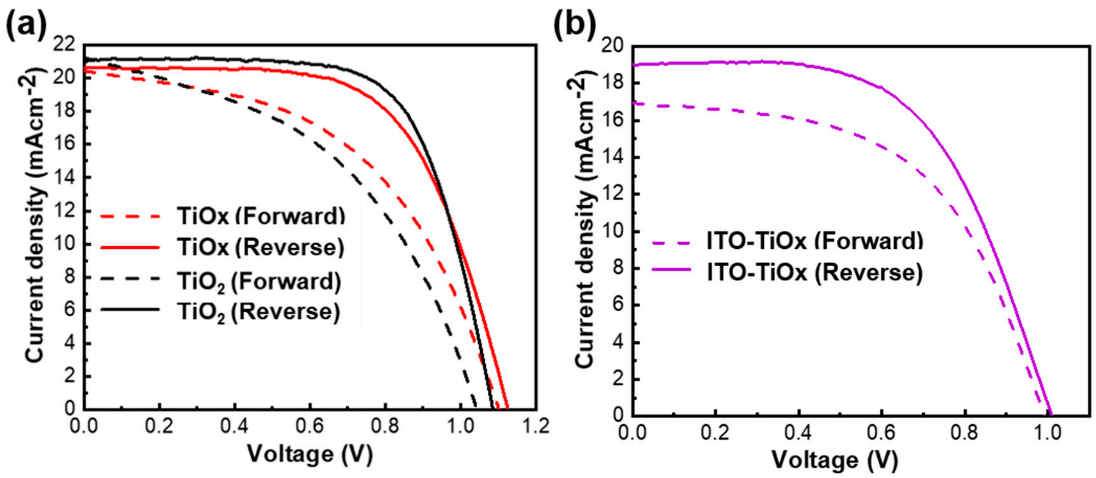

Figure S2. Forward Scan and Reverse Scan *J-V* curves of (a) TiO_x and TiO_2 films deposited on FTO-substrate; (b) TiO_x film grown on ITO-substrate.

Table S1. Nyquist plots fitting analysis parameters.

ETLs Layer	Rs (Ω)	R1(Ω)	CPE1-T	CPE1-P	R2(Ω)	CPE2-T	CPE2-P
TiOx	36.8	160.6	2.02E-8	1.05	123.5	3.05E-8	0.946
TiO_2	28.3	105.4	3.73E-8	1.02	116	5.26E-8	0.920

Table S2. Summary of device performance characteristics with TiO_x and TiO_2 based PSCs.

ETLs Layer	Scan direction	J_{SC} (mA/cm^2)	V_{oc} (V)	*FF*	PCE (%)
TiOx	Forward	20.44	1.10	0.50	11.21
	Reverse	20.64	1.12	0.63	14.51
TiO_2	Forward	21.21	1.04	0.46	10.12
	Reverse	21.06	1.08	0.68	15.50

Table S3. Summary of device performance characteristics with TiO_x film deposited on ITO-substrate based PSCs.

ETLs Layer	Scan direction	J_{SC} (mA/cm^2)	V_{oc} (V)	*FF*	PCE (%)
ITO-TiOx	Forward	16.94	0.99	0.54	9.13
	Reverse	19.02	1.01	0.58	11.13
